# Selective Detection of NO and NO_2_ with CNTs-Based Ionization Sensor Array

**DOI:** 10.3390/mi9070354

**Published:** 2018-07-16

**Authors:** Hui Song, Kun Li, Chang Wang

**Affiliations:** 1 School of Software Engineering, Qufu Normal University, Qufu 273165, China; 2 College of Engineering, Qufu Normal University, Rizhao 276825, China; 3 Department of Microelectronics, School of Electronics and Information Engineering, Xi’an Jiaotong University, Xi’an 710049, China; wangc369@126.com

**Keywords:** ionization sensor array, NO_x_, carbon nanotube (CNT), selectivity, non-self-sustaining discharge

## Abstract

The accurate detection of NO_x_ is an important issue, because nitrogen oxides are not only environmental pollutants, but also harm to human health. An array composed of two carbon nanotubes (CNTs)-based ionization sensors with different separations is proposed for NO and NO_2_ selective detection. The experimental results indicate that the CNTs-based ionization sensor has an intrinsic, monotonically decreasing response to NO or NO_2_. The sensor with 80 µm separations and 100 µm separations exhibited the highest sensitivity of −0.11 nA/ppm to 300 ppm NO and −0.49 nA /ppm to 70 ppm NO_2_, respectively. Although the effect of the NO_2_ concentration on the NO response is much stronger than that of NO on NO_2_, the array of these two sensors still exhibits the ability to simultaneously detect the concentrations of NO and NO_2_ in a gas mixture without component separation.

## 1. Introduction

The oxides of nitrogen, collectively termed as NO_x_ and mainly regarding NO + NO_2_, are major atmospheric pollutants. Anthropogenic emissions of NO_x_ are mostly from coal combustion and petroleum refining. Nitrogen oxides combine with water to form acid rain and with other pollutants to produce photochemical smog, which can greatly damage the environment. The accurate detection of NO_x_ is an important issue, because the nitrogen oxides are not only environmental pollutants, but also harm to human health. Many kinds of NO_x_ gas sensors have been investigated in recent years. In particular, nanostructured semiconducting metal oxides sensors [1,2,3,4,5] and carbon nanotubes (CNTs)-based sensors [6,7,8,9,10,11,12] have attracted much more concern. Due to the adsorption or desorption of gas molecules on the surface of metal oxides, the electrical conductivity of these sensors varies greatly with the changes in NO_x_ concentration. However, the metal oxides gas sensors have the limitations of requiring high operating temperatures, and in addition, the response of those sensors largely depends on the morphology of the metal oxides’ nanostructures [3].

A CNT, which has a large surface area to volume ratio and high electrical conductivity, can be an ideal candidate to operate at room temperature with high sensitivity [13]. One important use for CNTs is as a resistance sensor based on the change of electrical conductivity caused by gas adsorption and desorption. Almost all previously reported CNTs-based NO_x_ gas sensors are based on this mechanism [6,14,15,16,17]. Another important application of CNTs is in a carbon nanotube field effect transistor (CNFET), which has characteristics that are affected by gas-induced changes of contact properties and by the doping level of the nanotube [18,19]. However, higher cross-sensitivity and lower recovery times at room temperature are the major problems of these sensors [8,13,15]. Thus, the improvement of selectivity and response times is the primary concern of researchers. With respect to selectivity, Yao et al. introduced moisture to selectively detect NO_2_ and SO_2_ [6]. The resistance of the gas sensor decreased independent of the moisture levels in the case of NO_2_ and increased with SO_2_ exposure at a high humidity level. But whether this approach can selectively detect any other gases has not been reported. With respect to response times, Dojin Kim et al. proposed a high-performance conduction model for a nanostructured sensor, and they revealed that the maximum response occurs at a diameter near the complete depletion condition [12]. The model has been experimentally proven for a metal oxides nanostructure, but whether it is valid for carbon nanotubes requires future confirmation.

**Figure 1:**
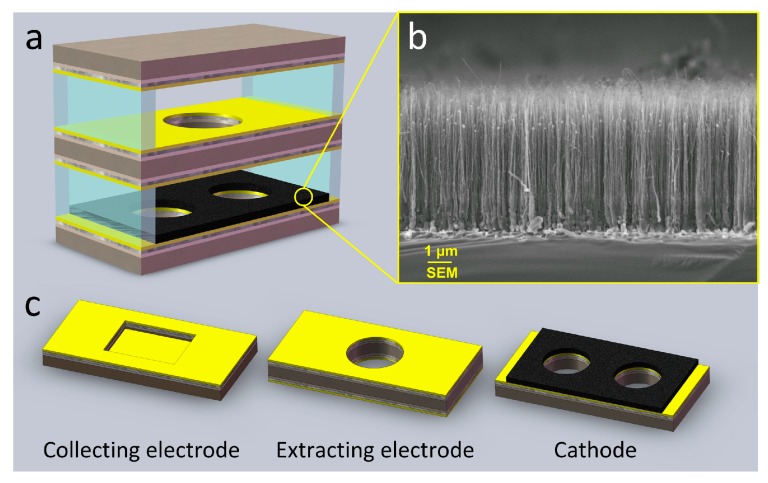
(**a**) Schematic diagram of the test system; (**b**) SEM image of the carbon nanotube (CNT) film; and (**c**) Schematic of the triple-electrode sensor structure.

**Figure 2:**
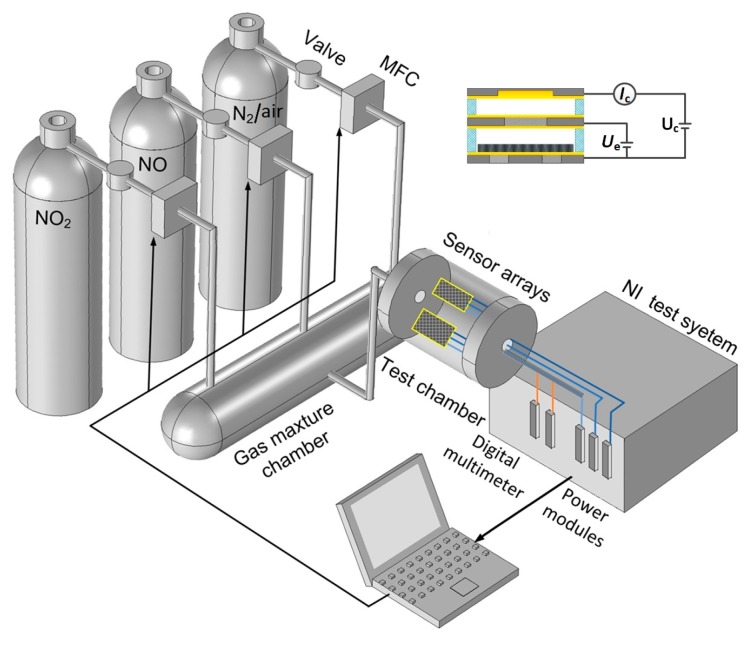
Schematic diagram of gas distribution and experimental testing system.

In this paper, we propose a triple-electrode ionization sensor with a CNT film cathode (Figure 1a) for NO or NO_2_ detection and an array consisting of two sensors with different electrode separations for simultaneously detecting a gas mixture of NO and NO_2_. The discharge of the CNT-based triple-electrode ionization sensor has been demonstrated to be non-self-sustaining when the appropriate extracting voltage *U*_e_ is applied [20,21]. We measure the NO_X_ concentration with a discharge current, which is determined by the gas concentration at a given electrode separation and under ambient conditions based on the Townsend discharge theory [22]. We also demonstrated that the array, which is composed of those CNT-based ionization sensors with different electrode separations (insert of Figure 2), has the ability to directly discriminate between any different type of gas without component separation. Here, we used the array of two sensors with 80 μm and 100 μm separations to simultaneously detect and distinguish NO and NO_2_. Gas sensing experiments were carried out at normal atmosphere and a relative humidity in the range of 21.1–21.9% RH. We also discuss the cross-sensitivity between the two gases.

## 2. Experimental Detail

### 2.1. Synthesis of CNT Films and Three Electrodes

For the growth of CNT films on the cathode, a piece of substrate coated with iron phthalocyanine (FeC_32_N_8_H_16_) is set in a tube furnace, which was preheated to 850 °C/890 °C with Ar/H_2_ flow, sequentially. Then, a quartz boat loaded with the annealed cathode was pushed into furnace, and the growth process immediately started. At 920 °C, iron phthalocyanine decomposed for about 13 min until the dark green smoke disappeared. The substrates were naturally cooled to room temperature under Ar exposure in the furnace. Then, vertically aligned carbon nanotube (CNT) film was grown by thermal chemical vapor deposition (TCVD) method. As can be seen from the transmission electron microscopy (FEI, Quanta 250 FEG, Hillsboro, OR, USA) images of the samples, as shown in Figure 1b, the CNT film is homogeneous and dense, and the length of the suspended CNTs is ~5–6 μm.

Three silicon slices in sizes of 27 mm × 8 mm × 450 μm were processed through a mask, photolithography, dry etching, and cleaning, resulting in a cathode with two cooling holes of 4 mm diameter, the extracting electrode with one 3 mm radial round hole, and the collecting electrode with a square blind rectangle of 8 × 6 mm^2^ in area and 200 μm in depth. Ti/Ni/Au films (50 nm/125 nm/400 nm) were sputtered in sequence on both sides of the extracting electrode, the inner side of the cathode, and the collecting electrode. Here, Ni film was used for preventing the inter-diffusion between the Ti and Au films. The three electrodes were rapidly annealed at 450 °C for about 50 s to enhance the bond strength between the substrate and the Ti/Ni/Au films (Figure 1c). Then, vertically aligned CNT film was transferred to the inner side of cathode with the wetting transfer method [23].

### 2.2. Fabrication of Sensors and Sensor Array

Polyester film, which is a good electrical insulating material due to its good insulation properties, exceptional thermal stability, and high impedance, was used to separate the cathode, the extracting electrode, and the collecting electrode. Polyester films with different thickness were cut to separate the electrodes. The gap distances between the cathode and the extracting electrode and between the extracting and the collecting electrodes were fixed as well (Figure 1a). Three electrodes with 100 µm separations were connected with three golden wires as the three pins of the sensor; thus, the NO_2_ sensor was fabricated in the same way as the NO sensor device but with 80 µm separations. Hence, the sensor array composed of these two sensors with 80 µm and 100 µm separations was finished.

### 2.3. Experimental Testing System

The stable voltages applied to the sensor array were supplied by power modules (NI PXI-4132, NI, Austin, TX, USA). When the concentration of NO (NO_2_) varied from 0 ppm to 1120 ppm (0–220 ppm), the collecting current was recorded by a precise digital multimeter (NI PXI-4071) controlled by a computer via the MXI interface of the test system (Figure 2).

The gas mixture was firstly prepared by mixing with dry air in the mixture chamber and then flowing it into the test chamber until atmospheric pressure was reached. The concentration of NO and NO_2_ were controlled by three mass flow controllers (MFC, Line Tech M3030 V with 1% accuracy, Line Tech, Inchon, Korea) with full scales of 100 mL/min for NO, 50 mL/min for NO_2_, and 1000 mL/min for dry air, respectively. Before each measurement, the test chamber was heated to 60 °C and pumped to a vacuum of ~5 kPa into which the well-mixed gas could flow. The gas sensing experiments were carried out at atmospheric pressure and a relative humidity of 20 ± 2% RH.

## 3. Results and Discussion

Because the applied extracting voltage *U*_e_ was lower than the breakdown voltage, our triple-electrode ionization gas sensor worked in a non-self-sustaining discharge state to reduce the damage to the CNTs caused by the electric breakdown. Thus, the non-self-sustaining discharge current could be used to measure the gas concentration with good stability, which had been demonstrated using the gold nanowires anode [24,25] and the CNTs-based cathode [26,27].

The electrons, emitted from the CNT-based cathode, collided with the gas molecules to produce more electrons and positive ions near the vicinity of the cathode, resulting in the occurrence of field ionization. A large number of positive ions migrated to the collecting electrode as collecting current *I_c_* through diffusion and drift under the opposite electrical field. Based on the theory of Townsend discharge, the collecting current *I_c_*, as part of the discharge current, was mainly determined by electrode separation and the first ionization coefficient *α* in the non-self-sustaining discharge at a given gas temperature as follows [28],

(1) $$I_{c}=I_{0}e^{\alpha d}$$

where *I*_0_ is the initial current, *d* is the electrode separation between the CNT-based cathode and the extracting electrode, and *α = APe^−BP/E^* is determined by the applied electric field *E* and partial pressure *P* of collision gases, because *A* and *B* are constants related to gas species and temperature. While *E* is proportional to the extracting voltage *U*_e_ at constant *d*, *P* is proportional to the gas concentration *φ* at constant gas temperature and volume. That is, the collecting current *I*_c_ is a function of the electrode separation *d*, the extracting voltage *U*_e_, the gas species, and the gas concentration φ. Thus, the array composed of two sensors with different electrode separations *d*_1_ and *d*_2_ could simultaneously detect the NO and NO_2_ concentration without component separation. The collecting currents *I_c_*_1_ and *I_c_*_2_ can be expressed as follow:

(2) $$I_{c1}=f(\varphi_{NO},\varphi_{NO_{2}},d_{1})$$

(3) $$I_{c2}=f(\varphi_{NO},\varphi_{NO_{2}},d_{2}).$$

By measuring the collecting currents $I_{c1}$ and $I_{c2}$, the gas concentrations $\varphi_{\mathrm{NO}}$ and $\varphi_{{\mathrm{NO}}_{2}}$ could be detected at a constant extracting voltage *U*_e_ and the given electrode separations. That is why we can use the sensor array to detect a gas mixture without any component separation.

**Figure 3:**
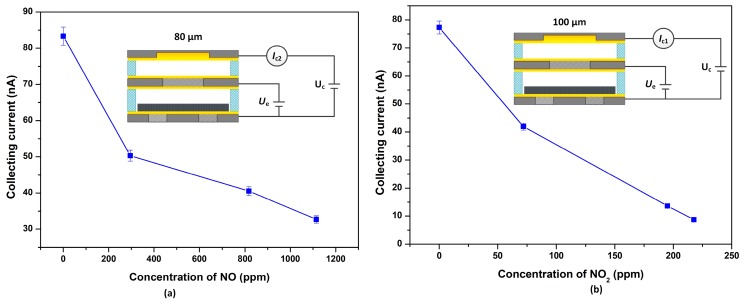
(**a**) NO response of the CNTs-based ionization sensor with 80 µm electrode separations and (**b**) NO_2_ response of the sensor with 100 µm separations at *U*_e_ = 150 V and *U*_c_ = 10 V.

The triple-electrode ionization sensors with 80 μm separations and 100 μm were are used to detect NO and NO_2_, respectively (insert of Figure 3a,b). The collecting currents decreased monotonically with the increasing of the NO and NO_2_ concentrations at a fixed *U*_c_ of 10 V and *U*_e_ of 150 V (Figure 3a,b). As the NO concentration increased from 0 to 1120 ppm, the collecting current decreased from 83.3 nA to 32.7 nA, and the sensor with 80 µm separations and 150 V extracting voltage exhibited the highest NO sensitivity of −0.11 nA/ppm to 300 ppm NO. For NO_2_, the collecting current decreased from 77.3 nA to 8.7 nA with an increasing NO_2_ concentration from 0 to 220 ppm, and the highest sensitivity was −0.49 nA/ppm to 70 ppm for the sensor with 100 µm separations at the 150 V extracting voltage. Here, the sensitivity *S* is defined as the ratio of the variation of the collecting current ∆*y* and the variation of gas concentration ∆*x*.

**Figure 4:**
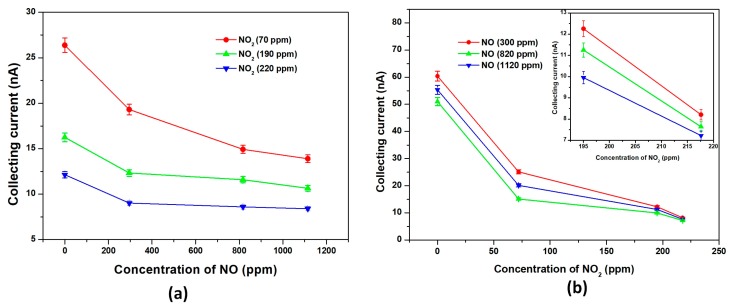
Simultaneous detection of NO and NO_2_ in a gas mixture using a sensor array with 80 μm and 100 μm separations at 150 V *U*_e_ and 10 V *U*_c_, respectively. The collecting currents decreased with increasing (**a**) NO and (**b**) NO_2_ concentrations in dry air.

Cross-sensitivity is a key problem in the practical application of gas selective detecting. Figure 4a,b show the response of each sensor in the array to the mixture of NO and NO_2_, respectively. The collecting current was reduced dramatically from 19.31 nA to 9.02 nA to 300 ppm NO with the NO_2_ concentration increasing from 70 ppm to 220 ppm (Figure 4a). The average variation of the collecting current for each NO concentration was about 10 nA and as high as half of its response. In other words, the CNTs-based ionization sensor with 80 µm separations could detect the concentration of NO very well no matter how the NO_2_ concentration in the gas mixture changed. While for NO_2_, the minimum variation in the collecting current was also nearly 400 pA and occurred at 220 ppm when the concentration of NO in the gas mixture changed (Figure 4b). Accordingly, the ionization sensor with 100 µm separations still had the ability to detect NO_2_ accurately in the gas mixture even though the effect of the NO concentration on the NO_2_ response was weaker (insert in Figure 4b).

In addition, all the NO response curves show almost the same shape at different NO_2_ concentrations (Figure 4a), and the same is true for NO_2_ (Figure 4b). This indicates that each sensor had intrinsic gas sensing properties. Moreover, the array was thought to show a good repeatability without considering the variation of the collecting current caused by changes in the concentrations of interference components. This was attributed to the good stability of the sensor due to the long life of the CNTs.

## 4. Conclusions

The non-self-sustaining discharge of the triple-electrode CNTs-based sensor makes it possible to measure gas concentration with the non-self-sustaining discharge current with good stability. The array of these sensors shows a monotonically decreasing response to NO_X_. The NO sensor with 80 μm separations and the NO_2_ sensor with 100 μm separations exhibited the highest sensitivity −0.11 nA/ppm to 300 ppm NO and −0.49 nA/ppm to 70 ppm NO_2_, respectively. The maximum variation in the collecting current of the NO sensor is about 10 nA, when the concentration of NO_2_ in mixture is changed, while the minimum variation of the NO_2_ sensor is also about 400 pA. Although NO_2_ had a stronger effect on NO than that of NO on NO_2_, the array of these two CNTs-based ionization sensors still has the ability to simultaneously detect the concentrations of NO_2_ and NO in a gas mixture without component separation. In addition, the response shape of each sensor is almost the same. This indicates that the array has intrinsic gas sensing properties and good stability.
